# Ganglion cell inner plexiform layer thickness measured by optical coherence tomography to predict visual outcome in chiasmal compression

**DOI:** 10.1038/s41598-022-17193-0

**Published:** 2022-09-01

**Authors:** Ga-In Lee, Joonhyoung Kim, Dongyoung Lee, Kyung-Ah Park, Sei Yeul Oh, Doo-Sik Kong, Sang Duk Hong

**Affiliations:** 1 Hangil Eye Hospital, Incheon, Korea; 2 Sungkyunkwan University School of Medicine, Seoul, Korea; 3 Department of Ophthalmology, Samsung Medical Center, Sungkyunkwan University School of Medicine, 81 Irwon-ro, Gangnam-gu, Seoul, 06351 South Korea; 4 Department of Neurosurgery, Endoscopic Skull Base Surgery Clinic, Brain Tumor Center, Samsung Medical Center, Sungkyunkwan University School of Medicine, Seoul, Korea; 5 Department of Otorhinolaryngology-Head and Neck Surgery, Samsung Medical Center, Sungkyunkwan University School of Medicine, Seoul, Korea

**Keywords:** Optic nerve diseases, CNS cancer

## Abstract

We evaluated the prognostic value of the preoperative macular ganglion cell inner plexiform layer (mGCIPL) thickness along with peripapillary retinal nerve fiber layer (pRNFL) thickness measured by optical coherence tomography (OCT) and estimated an optimal cut-off value to predict postoperative visual field (VF) recovery in adult patients with chiasmal compression after decompression surgery. Two hundred forty eyes of 240 patients aged 20 years or older for which preoperative high-definition Cirrus OCT parameters and pre- and postoperative visual function data were available. The prognostic power of pRNFL and mGCIPL thicknesses for complete postoperative VF recovery or significant VF improvement (improvement ≥ 2 dB in the mean deviation) were assessed. The cut-off values for OCT parameters for VF recovery were estimated. The study found that the higher the preoperative pRNFL and mGCIPL thicknesses, the higher the probability of complete postoperative VF recovery (*p* = 0.0378 and *p* = 0.0051, respectively) or significant VF improvement (*p* = 0.0436 and *p* = 0.0177, respectively). The area under the receiver operating characteristic analysis of preoperative OCT parameters demonstrated that the mGCIPL thickness showed an area under the curve (AUC) of more than 0.7 for complete VF recovery after decompression surgery (AUC = 0.725, 95% CI: 0.655, 0.795), and the optimal mGCIPL thickness cut-off value for complete VF recovery was 77.25 µm (sensitivity 69% and specificity 69%). Preoperative mGCIPL thickness was a powerful predictor of visual functional outcome after decompression surgery for chiasmal compression.

## Introduction

Chiasmal compression originates from stress in the optic chiasm due to various etiologies including pituitary adenoma, craniopharyngioma, meningioma, and vascular abnormalities^1–3^. It can commonly give rise to visual acuity or field dysfunction in specific patterns, which depends upon the location or type of the lesions and the degree of compression^3–5^. Previous reports have demonstrated the mechanisms of visual dysfunction in chiasmal compression, including conduction block, demyelination, ischemic insult, and retrograde and anterograde degeneration^6^. Axonal damage to retinal ganglion cells (RGCs) is caused by retrograde degeneration, spreading the injury from the optic chiasm toward the cell body in the retina^7^. The retrograde degeneration toward the RGCs due to compression of the visual pathway was corroborated in a study showing that osteopetrotic mutant mice with compressed optic canals had increased apoptotic cells in their retinas after birth^8^.

Since the advent of optical coherence tomography (OCT), noninvasive 2-dimensional cross-sectional imaging of the retina^9^, there have been many attempts to use OCT parameters to quantify the magnitude of retinal layer atrophy due to retrograde degeneration in chiasmal compression and predict postoperative visual outcomes^1,10–17^. To date, peripapillary retinal nerve fiber layer (pRNFL) thickness measured by OCT has been widely used as a prognostic factor in chiasmal compression after decompression surgery^1,10–15^. It was reported that the probability of near-normal visual functional improvement increased with increasing RNFL thickness up to approximately 85 µm, after which there were no further probability changes with increasing RNFL thickness^1^. In addition, after the automated quantification of the thickness of the retinal ganglion cell layer became available using high-resolution OCT, the association between macular ganglion cell layer thickness and vision prognosis was evaluated in several studies^16,17^. Ohkubo et al. reported that all ganglion cell complex parameters were significantly correlated with visual field (VF) parameters by observing 12 patients with chiasmal compression^16^. Another study showed that the mean VF deviation correlated better with ganglion cell complex thickness than RNFL thickness in 23 patients with chiasmal compression^17^.

The purpose of this study was to evaluate the prognostic value of preoperative macular ganglion cell and inner plexiform layer (mGCIPL) thicknesses along with pRNFL thickness measured by Cirrus high-definition OCT (HD-OCT) in a relatively large-scale study and newly estimate the statistical cut-off values for postoperative VF recovery in adult patients with chiasmal compression after decompression surgery.

## Results

This study included a total of 240 eyes of 240 patients with chiasmal compression. The mean follow-up duration after surgery was 8.85 ± 6.42 months (range, 4.0–34.31 months). One hundred eighty-eight patients had a pituitary adenoma, 21 patients had a craniopharyngioma, another 29 patients had a meningioma, and two patients had a Rathke’s cleft cyst, which resulted in chiasmal compression. The average age of the patients was 51 ± 14 years (range, 20–84 years) with men comprising 42.1% (101 patients) and women 57.9% (139 patients). The average spherical equivalent refractive errors (SER) value was −0.7 ± 1.7 diopters. The postoperative mean deviation (MD) of the VF (−3.9 ± 5.3 dB) was significantly improved compared to the preoperative MD of the VF (−8.6 ± 7.7 dB) (*p* < 0.0001). Also, the proportion of patients with normal postoperative best-corrected visual acuity (BCVA) (20/20) was 61.7% (148 patients), which was significantly increased compared to the proportion of patients with normal preoperative BCVA (40.0%, 96 patients) (*p* < 0.0001). In the postoperative magnetic resonance imaging (MRI) scans of this study population, 161 (67.1%) out of 240 patients had complete mass removal, 78 patients (32.5%) had a residual mass in any part (including very subtle residuals) after surgery, and one (0.42%) had residual masses still partially compressing the chiasm.

All patients were divided into two groups based on complete recovery and significant recovery of the VF. Based on the presence of complete VF recovery after decompression surgery, the complete recovery group was comprised of 61 patients (25.4%) and the partial or no recovery group was comprised of 179 patients (74.6%). The mean age of the group with complete recovery (46 years old) was younger than the group with partial or no recovery (52 years old) (*p* = 0.001). Older age was associated with a decreased probability of the complete recovery of VF defects. The odds of complete recovery were multiplied by 0.971 for each 1-year increase in age (odds ratio [OR] = 0.971; 95% confidence interval [CI]: 0.95–1.00; *p* = 0.0245). The group with complete recovery had better preoperative visual function including BCVA (*p* = 0.032) and MD of the VF (*p* < 0.001). The odds of complete recovery were multiplied by 1.112 for each 1-dB increase in the MD in VF testing ([Model 1] OR = 1.112, 95% CI: 1.04–1.19, *p* = 0.002; [Model 2] OR = 1.094, 95% CI: 1.02–1.17; *p* = 0.010). Also, the group with complete recovery had thicker preoperative pRNFLs and mGCIPLs than the group with partial or no recovery (pRNFL, *p* < 0.001; mGCIPL, *p* < 0.001) (Table 1). The odds of complete recovery were multiplied by 1.033 and 1.068 for each 1-μm increase in pRNFL and mGCIPL thickness, respectively (pRNFL, OR = 1.033, 95% CI: 1.00–1.07, *p* = 0.0378; mGCIPL, OR = 1.068, 95% CI, 1.02–1.12, *p* = 0.0051) (Table 2).

**Table 1:** Demographics of patients with chiasmal compression based on complete and significant recovery.

	Stratified by complete recovery			Stratified by significant recovery		
	Complete recovery	Partial or no recovery	*P*-value	Significant recovery	No significant recovery	*P*-value
Number	61	179		148	92	
**Sex (%)**						
Female	37 (60.7)	102 (57.0)	0.725^a^	89 (60.1)	50 (54.3)	0.454^a^
Male	24 (39.3)	77 (43.0)		59 (39.9)	42 (45.7)	
Age (years)	46 ± 13	52 ± 14	**0.001**^**b**^	51 ± 14	50 ± 15	0.978^b^
**Preoperative BCVA [logMAR] (%)**						
20/20	32 (52.5)	64 (35.8)	**0.032**^**a**^	44 (29.7)	52 (56.5)	** < 0.001**^**a**^
Less than 20/20	29 (47.5)	115 (64.2)		104 (70.3)	40 (43.5)	
**Postoperative BCVA [logMAR] (%)**						
20/20	46 (75.4)	102 (57.0)	**0.016**^**a**^	91 (61.5)	57 (62.0)	1.000^a^
Less than 20/20	15 (24.6)	77 (43.0)		57 (38.5)	35 (38.0)	
SER	−1.0 ± 1.4	−0.6 ± 1.8	**0.070**^**b**^	−0.8 ± 1.8	−0.6 ± 1.5	0.440^b^
Preoperative VFMD (dB)	−4.8 ± 4.2	−9.9 ± 8.1	** < 0.001**^**b**^	−11.7 ± 7.6	−3.7 ± 4.6	** < 0.001**^**b**^
Postoperative VFMD (dB)	−0.3 ± 0.8	−5.1 ± 5.7	** < 0.001**^**b**^	−3.7 ± 5.0	−4.2 ± 5.9	0.749^b^
pRNFL thickness (μm)	93.9 ± 10.1	85.7 ± 13.8	** < 0.001**^**b**^	85.9 ± 12.6	90.9 ± 14.1	**0.001**^**b**^
mGCIPL thickness (μm)	79.1 ± 6.8	71.5 ± 10.4	** < 0.001**^**b**^	71.6 ± 9.9	76.4 ± 10.0	** < 0.001**^**b**^

*BCVA* best-corrected visual acuity, *SER* spherical equivalent refractive errors, *VFMD* visual field mean deviation, *pRNFL* peripapillary retinal nerve fiber layer, *mGCIPL* macular ganglion cell-inner plexiform layer.

Significant values are indicated in bold.

^a^Chi-squared test.

^b^Wilcoxon rank sum test.

**Table 2:** Multivariable logistic regression analysis of complete recovery.

Parameter	Model 1^a^						Model 2^a^					
	Estimate	Standard error	*P* value	Odds ratio	95% CI		Estimate	Standard error	*P* value	Odds ratio	95% CI	
Age	−0.0292	0.013	**0.0245**	**0.971**	**0.947**	**0.996**	−0.0278	**0.0132**	**0.0348**	**0.973**	**0.948**	**0.998**
Preoperative BCVA	0.1209	0.3495	0.7295	1.128	0.569	2.239	0.2751	0.3533	0.4362	1.317	0.659	2.632
SER	−0.0903	0.1076	0.4014	0.914	0.74	1.128	−0.1071	0.1094	0.3276	0.898	0.725	1.113
Preoperative VFMD	0.1065	0.0345	**0.002**	**1.112**	**1.04**	**1.19**	0.0899	**0.0350**	**0.0102**	**1.094**	**1.022**	**1.172**
pRNFL thickness	0.0327	0.0157	**0.0378**	**1.033**	**1.002**	**1.066**	NA					
mGCIPL thickness	NA						0.0659	**0.0235**	**0.0051**	**1.068**	**1.020**	**1.119**

Significant values are indicated in bold.

*BCVA* best-corrected visual acuity, *CI* confidence interval, *SER* spherical equivalent refractive errors, *VFMD* visual field mean deviation, *pRNFL* peripapillary retinal nerve fiber layer, *mGCIPL* macular ganglion cell-inner plexiform layer, *NA* Not applicable.

^a^As the peripapillary retinal nerve fiber layer and macular ganglion cell-inner plexiform layer thicknesses are highly anatomically related, we analyzed them by dividing them into two models (Model 1 and 2).

The group with significant VF recovery of at least 2 dB or more included 148 patients (61.7%), and the group with no significant VF recovery was comprised of 92 patients (38.3%). Thirty-eight patients with a preoperative MD of the VF of greater than -2 dB (range, −1.97 dB to −0.05 dB) were also included in the no significant VF recovery group. There was no statistically significant difference in the mean age between the group with significant recovery (51 years old) and the group without significant recovery (50 years old) (*p* = 0.978). The mean preoperative logMAR visual acuity (*p* < 0.001) and MD of the VF (*p* < 0.001) in the group with significant recovery were relatively worse than in the group without significant recovery. The odds of significant recovery were multiplied by 0.753 for each 1-dB decrease in MD ([Model 1] OR = 0.753, 95% CI: 0.69–0.82; *p* < 0.0001; [Model 2] OR = 0.742; 95% CI: 0.68–0.82; *p* < 0.0001). The mean preoperative pRNFL and mGCIPL thicknesses in the group with significant recovery were relatively less than those in the group without significant recovery (pRNFL, *p* = 0.001; mGCIPL, *p* < 0.001) (Table 1). However, the odds of significant recovery were multiplied by 1.032 and 1.053 for each 1-μm increase in the pRNFL and mGCIPL, respectively (pRNFL, OR = 1.032; 95% CI: 1.00–1.06; *p* = 0.0436; mGCIPL, OR = 1.053; 95% CI: 1.01–1.10; *p* = 0.0177) (Table 3).

**Table 3:** Multivariable logistic regression analysis of significant recovery.

Parameter	Model 1^a^						Model 2^a^					
	Estimate	Standard error	*P* value	Odds ratio	95% CI		Estimate	Standard error	*P* value	Odds ratio	95% CI	
Preoperative BCVA	0.1406	0.3341	0.6739	1.151	0.598	2.215	0.2762	0.3380	0.4137	1.318	0.680	2.556
Preoperative VFMD	−0.2834	0.0460	** < 0.0001**	**0.753**	**0.688**	**0.824**	−0.2981	**0.0483**	** < 0.0001**	**0.742**	**0.675**	**0.816**
pRNFL thickness	0.0311	0.0154	**0.0436**	**1.032**	**1.001**	**1.063**	NA					
mGCIPL thickness	NA						0.0514	**0.0217**	**0.0177**	**1.053**	**1.009**	**1.099**

Significant values are indicated in bold.

*BCVA* best-corrected visual acuity, *CI* confidence interval, *SER* spherical equivalent refractive errors, *VFMD* visual field mean deviation, *pRNFL* peripapillary retinal nerve fiber layer, *mGCIPL* macular ganglion cell-inner plexiform layer, *NA* not applicable.

^a^As the peripapillary retinal nerve fiber layer and macular ganglion cell-inner plexiform layer thicknesses are highly anatomically related, we analyzed them by dividing them into two models (Model 1 and 2).

**Figure 1:**
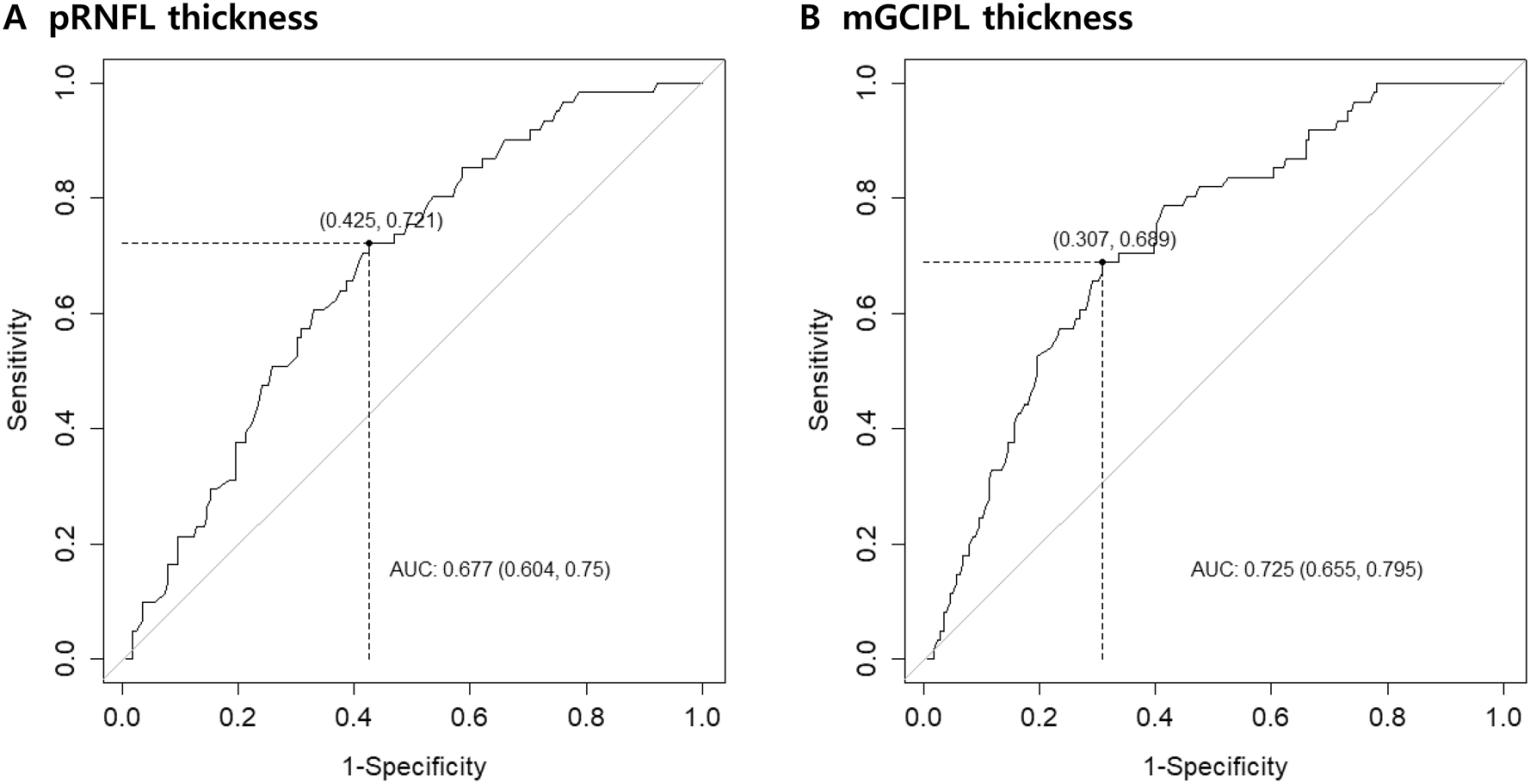
Receiver operating characteristics curves based on the analysis of (**A**) peripapillary retinal nerve fiber layer and (**B**) macular ganglion cell-inner plexiform layer thickness, distinguishing eyes with complete visual field recovery from those with preoperative visual field defects. The sensitivity (with 95% confidence intervals) at a specificity of 95% is indicated for each curve.

To determine the optimal OCT parameter cut-off values, the receiver operating characteristic (ROC) curves of the pRNFL and mGCIPL for complete VF recovery (Fig. 1) and significant VF recovery were obtained. Although there was no significant difference between the pRNFL thickness (areas under the ROC curve [AUC] 0.677) and mGCIPL thickness (AUC 0.726) in the ability to predict complete recovery (*p* = 0.0695), the optimal cut-off value for a complete VF recovery was derived from only an mGCIPL thickness of 77.25 μm (sensitivity 69% and specificity 69%) with an AUC of more than 0.7. Regarding significant recovery, there was no significant difference between the pRNFL and mGCIPL thicknesses in the ability to predict significant recovery (*p* = 0.3601). The optimal cut-off values for pRNFL and mGCIPL thicknesses for a significant VF recovery could not be derived as the AUC was less than 0.7.

## Discussion

In this study, both the preoperative pRNFL and mGCIPL thicknesses appeared to be associated with complete VF recovery, which was consistent with previous studies^1,10,13,16–18^. Previously, Danesh-Meyer et al. suggested that pRNFL thickness might serve as a prognostic indicator that correlated with final visual outcomes after surgical decompression^18^. The authors explained that the pRNFL demonstrated on OCT reflected the surviving functional axons, and thereby, it had the potential to predict VF improvement after decompression surgery^18^. It was verified by the finding in another report that the patients in the normal pRNFL thickness group had a larger magnitude of VF improvement than the thin pRNFL group^1^. Jacob et al. also reported that the odds of complete VF recovery were multiplied by 1.29 for each 1-µm increment in mean RNFL thickness in 19 patients with pituitary adenomas^10^. In the present study, the odds of complete VF recovery were multiplied by 1.03 for each 1- µm increase in mean pRNFL thickness. Danesh-Meyer et al. previously reported that the cut-off value for a significant VF recovery of > 10 dB was 80 µm in their study subjects^1^. They also reported that eyes with a thicker pRNFL were more likely to achieve complete VF recovery until a pRNFL thickness of 85 µm, after which there was no added benefit of having a thicker pRNFL^1^. In this study, the optimal cut-off value for a complete VF recovery could not be derived statistically for pRNFL thickness but it could be derived for mGCIPL thickness. The discrepancy in the results regarding the cut-off values between the studies could be due to the different statistical analysis methods, different characteristics of the study subjects, and different criteria for visual recovery. We used the same criterion for significant VF recovery as that of Danesh-Meyer et al., which was an improvement of 2 dB or more on the VF test^1^, but used different criteria for complete VF recovery, following the criteria of Ohkubo et al.^16^, which was the absence of clusters of three or more non-edge points with *p* < 0.05 and no point with *p* < 0.01 in the pattern deviation probability plot, a PSD within the 95% confidence limits, and a glaucoma hemifield test result within the normal limits. We also used Cirrus OCT in the analysis, which was different from the Stratus OCT used in the previous study, which may have caused a difference in the values.

The thickness of the ganglion cell complex (GCC), which consists of the nerve fiber layer, ganglion cell layer, and inner plexiform layer, measured by OCT was suggested as another powerful predictive tool for visual recovery in chiasmal compression in previous studies of 12 patients^16^ and 23 patients with pituitary tumors^17^. Both studies reported that GCC thickness was significantly correlated with VF outcomes in chiasmal compression^16,17^. Our study results also showed a positive association between preoperative mGCIPL thickness and visual prognosis. In the present study, the odds of complete VF recovery were multiplied by 1.07 for each 1-µm increase in mean mGCIPL thickness. In addition, we newly estimated cut-off values for the mGCIPL for visual recovery in chiasmal compression in this study. The approximate cut-off value for the mGCIPL for complete VF recovery was 77.25 μm (sensitivity 69% and specificity 69%). Setting cut-off values for the OCT parameters to predict visual outcomes can be helpful in counseling patients, determining the timing of the surgery, and planning postoperative management strategy. These results suggest the possibility that mGCIPL thickness may play an important role in predicting the visual outcomes.

To date, along with OCT parameters, various factors, including age^19,20^ and preoperative VF defects^21,22^, have also been reported to be associated with the recovery of visual function in chiasmal compression. Previously, Dutta et al. reported that increasing age was related to poorer final visual outcomes in patients with pituitary adenomas, and the reason was explained by the decreased capability for neuronal regeneration^19^. Similarly, Barzaghi et al. reported that the VF outcomes of patients over 50 years old were worse than in younger patients after decompression surgery and that young age was an independent positive prognostic factor^20^. The authors explained that younger patients are probably more able to compensate for microvascular deprivation due to the tumor compression of small vessels^20^. Regarding the preoperative VF as a prognostic factor, Gnanalingham et al. reported that the only independent predictor of the postoperative recovery of the VF was the degree of preoperative VF deficit while none of the other factors including age, symptom duration, the operating surgeon, the presence of optic atrophy, preoperative visual acuity, tumor extent, or postoperative radiotherapy had a significant influence on VF recovery in their study of 41 patients with pituitary adenomas^21^. Lee et al. also reported that worse preoperative VF defects were significantly associated with worse visual outcomes^22^. This study also showed that age, preoperative VF defects, and inner-retinal layer thicknesses were significantly associated with complete VF recovery, supporting the results of the previous studies. One encouraging result of this study was that when the criterion was significant VF recovery, age did not significantly affect the probability of recovery. Thus, based on the results, the probability of a complete VF recovery decreased but the probability of obtaining significant VF recovery did not significantly change in elderly patients, suggesting that both in younger and elderly patients, visual benefits can be expected through active intervention.

There were several limitations to this study. The study was conducted using a retrospective design, and thereby the follow-up period of each patient varied. Also, we recruited only Korean patients. Therefore, the direct application of these data to other ethnicities may need to be qualified.

In this study, we demonstrated a significant association between postoperative visual recovery after decompression surgery and preoperative OCT parameters including pRNFL thickness and mGCIPL thickness in patients with chiasmal compression. The optimal cut-off value in OCT parameters for complete VF recovery after decompression surgery could be derived from the mGCIPL thickness with an estimated cut-off value of 77.25 µm, sensitivity of 69%, and specificity of 69%. Preoperative mGCIPL thickness may serve as a valuable predictor for visual functional outcomes after decompression surgery in patients with chiasmal compression.

## Methods

This retrospective longitudinal study included 240 eyes of 240 patients aged 20 years or more with chiasmal compression at the Neuro-ophthalmology and Neurosurgery Department of Samsung Medical Center between March 2018 and June 2020. This study was conducted according to the tenets of the Declaration of Helsinki. The Institutional Review Board of Samsung Medical Center (Seoul, Republic of Korea) approved this study and waived the requirement for informed consent for patients with chiasmal compression. The diagnosis of chiasmal compression was based on preoperative VF defects and/or visual acuity and MRI scans evidence of tumor compression of the optic chiasm for all the patients in this study. Patients with other ophthalmic diseases (glaucoma, a refractive error greater than 6.0 diopters of spherical equivalent to high myopia and hyperopia, or 3.0 diopters of astigmatism, amblyopia, retinal diseases, or other optic neuropathy) and previous retinal surgery that affected the thickness of the intra-retinal layer were excluded. Patients diagnosed with known systematic or inflammatory diseases such as cancer and multiple sclerosis were also excluded. Patients who showed reliable VF results (≤ 33% false-positive and false-negative results; fixation losses < 20%) were included while patients with unreliable VF results were excluded. All patients underwent trans-sphenoidal tumor resection and visited the ophthalmoogy and neurosurgery clinic preoperatively and 4–6 and 6–12 months postoperatively, and annually thereafter. BCVA, VF test results, Cirrus OCT measurements before surgery, and the BCVA and VF test results at the latest visit after surgery were collected and analyzed in this study.

To assess the efficacy of chiasmal decompression after surgery, postoperative MRI was routinely performed. The neurosurgeons and radiologists also routinely met in a neurosurgery conference to evaluate the postoperative MRI results. The patients underwent ophthalmic examinations including BCVA, visual field testing, and HD-OCT pre- and postoperatively. The BCVA was converted to a logarithm of the minimum angle of resolution (logMAR).

**Figure 2:**
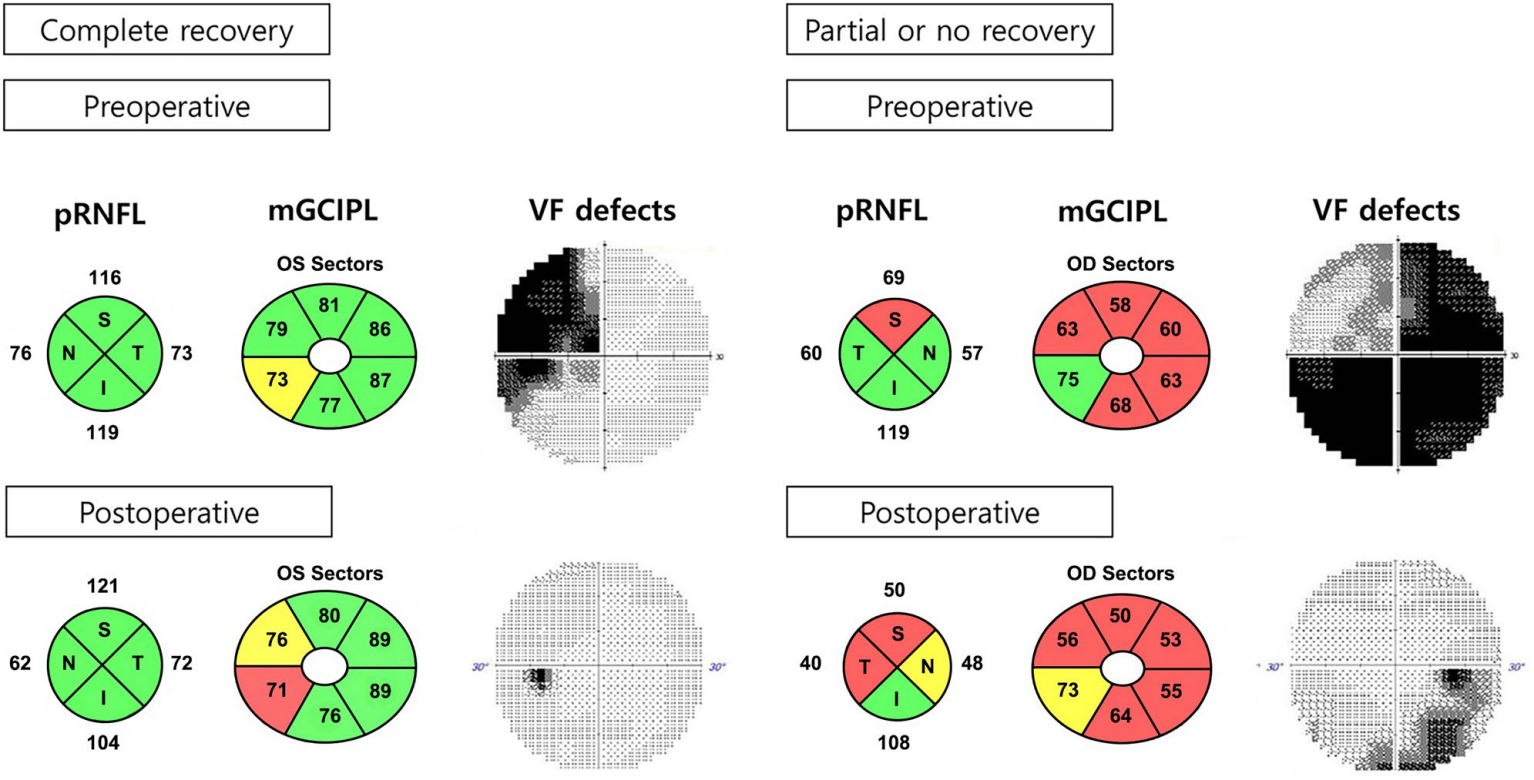
Representative cases in the complete recovery group and the partial or no recovery group of patients with chiasmal compression (both patients showed significant visual field [VF] recovery ≥ 2 dB in the mean deviation). Peripapillary retinal nerve fiber layer (pRNFL) and macular ganglion cell-inner plexiform layer (mGCIPL) thicknesses and VF defects are presented in each group pre- and postoperatively.

The patients were divided into two groups based on two criteria. One criterion was the complete recovery of VF defects versus partial or no recovery from preoperative VF defects. A complete recovery was defined by the absence of clusters of three or more non-edge points with *p* < 0.05 and no point with *p* < 0.01 in the pattern deviation probability plot, a pattern standard deviation (PSD) within the 95% confidence limits, and a glaucoma hemifield test result within the normal limits (Fig. 2)^16^. The other criterion was the significant recovery of VF defects versus no significant recovery. Significant recovery was defined by an improvement in the VF of ≥ 2 dB from the preoperative baseline^1^.

All included patients were scanned with a Cirrus HD-OCT (Carl Zeiss Meditec AG, Jena, Germany). The pRNFL thickness was measured using the optic disc cube 200 × 200 protocol with Cirrus software. A recognition algorithm detected the inner and outer borders of the pRNFL. This protocol generated a cube of data via a 6-mm-square grid. A circle with a diameter of 3.46 mm was automatically centered on the optic disc. This analytical protocol yielded the average pRNFL thickness, mapped four quadrants (superior, inferior, nasal, and temporal), and classified the results compared to an internal normative database. Only scans with a signal strength of ≥ 6 without motion artifacts were included. Using the macular ganglion cell analysis algorithm, the thickness of the mGCIPL was evaluated. The thickness of the mGCIPL was automatically measured at various locations around the fovea (superonasal, superior, superotemporal, inferonasal, inferior, and inferotemporal). The superotemporal and inferotemporal values were averaged and calculated as the value of the temporal area and superonasal and inferonasal values were averaged and calculated as the value of the nasal area. The average value of the four quadrants was used in the statistical analysis.

Only a single eye (the one with the worse VF defect based on the MD of each patient with chiasmal compression) was selected for analysis.

The VF perimetry of each patient was determined with a Humphrey Field Analyzer using the 30-2 Swedish interactive thresholding algorithm-standard protocol (Humphrey 740 Visual Field Analyzer, Carl Zeiss Meditec Inc. Dublin, CA, USA). Only reliable VFs (≤ 33% false positives and false negatives; fixation losses < 20%) were used in the study. The MD was used for the analysis.

### Statistical analysis

Continuous and categorical variables are presented as the mean ± standard deviation (SD) and frequency (percentage), respectively. For univariable analysis, the Chi-squared test was used to compare gender and BCVA, which was categorized based on 20/20 or less. The Wilcoxon rank-sum test was used to compare age, SER, the pre- and postoperative MD of the VF, and the preoperative average pRNFL and mGCIPL thickness values between the patients with chiasmal compression based on complete recovery and the significant recovery of VF defects. After selecting the variables with a *p*-value of < 0.1 in univariable analysis, the logistic regression model was used to test the association between a complete recovery or the significant recovery of VF and associated factors by multivariable analysis. Multicollinearity using the variance inflation factor (VIF) was assessed. There were no variables with a VIF of < 4. As the pRNFL and mGCIPL thicknesses are highly anatomically related, we analyzed them by dividing them into two models (Models 1 and 2). The ROC curve analysis was performed to evaluate the ability of the pRNFL and mGCIPL thicknesses to predict complete recovery or significant recovery of the VF. For the preoperative mGCIPL thicknesses, which had AUC greater than 0.7^23^, an optimal cutoff value was estimated using Youden’s Index^24^. To compare the AUCs of pRNFL and mGCIPL thicknesses for complete recovery and significant recovery, DeLong’s test was performed^25^. Bonferroni’s correction was applied to the *p*-value due to multiple testings. A *p*-value of less than 0.05 was considered statistically significant. All statistical analyses were performed with SAS version 9.4 (SAS Institute, Cary NC, USA).
